# Venovenous vs. Venoarterial Extracorporeal Membrane Oxygenation in Infection-Associated Severe Pediatric Acute Respiratory Distress Syndrome: A Prospective Multicenter Cohort Study

**DOI:** 10.3389/fped.2022.832776

**Published:** 2022-03-18

**Authors:** Yun Cui, Yucai Zhang, Jiaying Dou, Jingyi Shi, Zhe Zhao, Zhen Zhang, Yingfu Chen, Chao Cheng, Desheng Zhu, Xueli Quan, Xuemei Zhu, Wenyan Huang

**Affiliations:** ^1^Department of Critical Care Medicine, Shanghai Children's Hospital, School of Medicine, Shanghai Jiao Tong University, Shanghai, China; ^2^Institute of Pediatric Critical Care, Shanghai Jiao Tong University, Shanghai, China; ^3^Pediatric Intensive Care Unit, Senior Department of Pediatrics, The Seventh Medical Center of Chinese People's Liberation Army General Hospital, Beijing, China; ^4^Pediatric Intensive Care Unit, First Hospital of Jilin University, Changchun, China; ^5^Critical Care Medicine, Children's Hospital Affiliated to Chongqing Medical University, Chongqing, China; ^6^Pediatric Intensive Care Unit, Shengjing Hospital Affiliated to China Medical University, Shenyang, China; ^7^Department of Critical Care Medicine, Hunan Children's Hospital, Changsha, China; ^8^Surgical Intensive Care Unit of Henan Children's Hospital, Children's Hospital Affiliated to Zhengzhou University, Zhengzhou, China; ^9^Critical Care Medicine, Children's Hospital Affiliated to Fudan University, Shanghai, China; ^10^Department of Nephrology and Rheumatology, Shanghai Children's Hospital, School of Medicine, Shanghai Jiao Tong University, Shanghai, China

**Keywords:** venovenous, venoarterial, ECMO, PARDS, mortality, complications

## Abstract

**Background:**

Extracorporeal membrane oxygenation (ECMO) has been increasingly used as rescue therapy for severe pediatric acute respiratory distress syndrome (PARDS) over the past decade. However, a contemporary comparison of venovenous (VV) and venoarterial (VA) ECMO in PARDS has yet to be well described. Therefore, the objective of our study was to assess the difference between VV and VA ECMO in efficacy and safety for infection-associated severe PARDS patients.

**Methods:**

This prospective multicenter cohort study included patients with infection-associated severe PARDS who received VV or VA ECMO in pediatric intensive care units (PICUs) of eight university hospitals in China between December 2018 to June 2021. The primary outcome was in-hospital mortality. Secondary outcomes included ECMO weaning rate, duration of ECMO and mechanical ventilation (MV), ECMO-related complications, and hospitalization costs.

**Results:**

A total of 94 patients with 26 (27.66%) VV ECMO and 68 (72.34%) VA ECMO were enrolled. Compared to the VA ECMO patients, VV ECMO patients displayed a significantly lower in-hospital mortality (50 vs. 26.92%, *p* = 0.044) and proportion of neurologic complications, shorter duration of ECMO and MV, but the rate of successfully weaned from ECMO, bleeding, bloodstream infection complications and pump failure were similar. By contrast, oxygenator failure was more frequent in patients receiving VV ECMO. No significant intergroup difference was observed for the hospitalization costs.

**Conclusion:**

These positive findings showed the conferred survival advantage and safety of VV ECMO compared with VA ECMO, suggesting that VV ECMO may be an effective initial treatment for patients with infection-associated severe PARDS.

## Introduction

Pediatric acute respiratory distress syndrome (PARDS) is currently defined as the presence of hypoxia in the context of a new lung infiltrate occurring within 7 days of a known insult (1). Multiple triggers for PARDS have been identified, including infections, direct lung injury, aspiration of gastric contents, and other critical clinical conditions (2–4). However, infection (including pneumonia and sepsis) remains the leading cause of PARDS in several studies (5–11). Severe PARDS is defined as PARDS with severe hypoxemia [oxygenation index (OI) > 16] according to Pediatric Acute Lung Injury Consensus Conference (PALICC) (1). Overall, the mortality rate of PARDS is around 15%, while the mortality rate for severe PARDS ranges from 20 to 40% (1, 3, 5, 12, 13). Extracorporeal membrane oxygenation (ECMO) is a modified form of the pulmonary or cardiopulmonary bypass that may support severe respiratory failure, including PARDS. The Extracorporeal Life Support Organization (ELSO) database, the largest registry database specifically based on ECMO course data, shows that the use of ECMO for pediatric respiratory failure has expanded rapidly in recent years (14).

There are two ECMO modalities:. venovenous (VV) and venoarterial (VA). Once the decision has been made to initiate ECMO support for the patients with PARDS, the subsequent determination is the choice of ECMO modality. VV ECMO drains blood from the venous system, reinfuses blood into the venous system, and provides gas exchange but no direct cardiac support. In contrast, VA ECMO drains blood from the venous system, reinfuses into the arterial system, and provides complete cardiopulmonary support. However, critical cardiac benefits indirectly result from the initiation of VV ECMO to correct hypercarbia and acidosis, and decrease in right ventricular afterload. Furthermore, the inherent risks of VA ECMO should be considered. Compared to VV ECMO, VA ECMO was independently associated with an increased risk of neurologic injury, stroke and bleeding in neonatal and pediatric studies (15–17). Therefore, VV ECMO has become the more commonly used for respiratory failure refractory in pediatric critical care (18). With the steadily increasing use of ECMO, there is an increasing need to identify the ideal support modality. A review of the ELSO database in adults with acute respiratory distress syndrome (ARDS) concluded that VV ECMO was associated with a higher survival rate to discharge but similar complication rates compared with VA ECMO (19). Similarly, a study of neonatal respiratory failure demonstrated that VV ECMO was safe and effective for the treatment, even in the presence of significant inotropic support (20). However, to date, there is a lack of studies to compare the efficacy and safety of VV and VA ECMO in PARDS.

The study aimed to assess the difference between VV and VA ECMO in efficacy and safety for infection-associated severe PARDS patients.

## Materials and Methods

### Study Design

We designed and implemented a prospective multicenter cohort study. The ethics committee approved the study protocol (2018R052-F01). In addition, each participating institution obtained institutional review board approval and informed consent from the legal guardian of participating patients. This study followed the Reporting of Observational Studies in Epidemiology (STROBE) guideline. This study has registered in the Chinese Clinical Trial Registry, and the registry number is ChiCTR1800019555.

### Setting and Participants

In pediatric intensive care units (PICUs) of eight university hospitals in China, patients with infection-associated severe PARDS who received VV or VA ECMO between December 2018 to June 2021 were enrolled. Femoral vessels are not adequate for venous drainage or arterial return until approximately 15 kg or 2 years of age. Furthermore, VV ECMO cannot be considered for patients aged <2 years due to the lack of double-lumen cannulas for VV ECMO in China. Thus, we enrolled patients aged from 2 to 18 years, fulfilled the definition of severe ARDS with both PALICC (OI ≥ 16) and Berlin criteria (PaO_2_/FiO_2_ <100 mmHg) (21, 22), and met the ECMO to Rescue Lung Injury in Severe ARDS (EOLIA) criteria for initiation of ECMO (23). Severe infection was defined as an individual with a Pediatric Sequential Organ Failure Assessment (pSOFA) score ≥ 2 at admission to the PICU or an increase in the pSOFA score of ≥2 in 24 h (24). Severe PARDS was identified based on hypoxemia severity (OI ≥ 16 and PaO_2_/FiO_2_ <100 mmHg) (21).

Exclusion criteria included the following: (1) history of heparin-induced thrombocytopenia, (2) cancer with a life expectancy of fewer than 5 years, (3) long-term chronic respiratory insufficiency treated with oxygen therapy or noninvasive ventilation, (4) irreversible neurologic injury, (5) an expected difficulty in obtaining vascular access for ECMO in the femoral or jugular vein, (6) cardiac failure and refractory shock resulting in VA ECMO, (7) a decision to withhold or withdraw life-sustaining therapies, and (8) a situation if death was deemed imminent by the treating pediatric intensivist at the time of screening.

### Outcome and Variables

Epidemiological and clinical characteristics of study participants were documented in a case report form (CRF). Demographic details and pre-ECMO characteristics included: age, gender, comorbidities, infection site, pathogen, pre-ECMO rescue therapy, ventilation parameters, blood gas, Pediatric Risk of Mortality Score III (PRISM III) scores, Pediatric Index of Mortality 3 (PIM3), SOFA score, and Vasoactive-Inotropic Score (VIS). The primary outcome was in-hospital mortality. Secondary outcomes included ECMO weaning rate, duration of ECMO and mechanical ventilation (MV), PICU, and hospital lengths of stay, ECMO-related complications (including hemorrhagic, neurological, infectious, or mechanical), and hospitalization costs. Oxygenator failure was defined as ECMO patients that required a system exchange due to worsened gas transfer or device-induced coagulation disorder of the membrane oxygenators (25). All patients were followed up within 6 months of discharge. Patients were grouped based on initial cannulation strategy to either VV or VA ECMO, and the demographic details, pre-ECMO characteristics, ECMO settings, and the outcome were compared between groups. In order to evaluate the robustness of results, the sensitivity analyses were performed, including the 1 and 3-month mortality post ECMO weaning.

### VV and VA ECMO Cannulation Techniques and Management

Due to the lack of double-lumen cannulas for VV ECMO in China, all participator centers selected multisite configuration with single-lumen ECMO catheters for VV and VA ECMO at the patient's bedside in the PICUs. Among eight participator PICUs, only three enrolling centers can implant either VA or VV ECMO by surgical or percutaneous peripheral cannulation; other five centers can perform surgical VA ECMO only (mainly via the right internal jugular vein and the right carotid artery access). Consequently, VV or VA cannulation procedures were selected based on the patient's hemodynamic status, the size of femoral arteriovenous vessels, as well as the center's experience. Furthermore, for patients with a primary respiratory indication for ECMO and appropriate size of femoral venous vessels, we preferentially use VV ECMO as the initial treatment modality. Echocardiography was performed in all patients before ECMO cannulation.

The titration strategy and anticoagulation management were standardized across all centers. Unfractionated heparin was given as a bolus (75 units per kilogram) at cannulation, followed by an initial maintenance dose of 20 U/kg/h during ECMO. Heparin maintenance dose was adjusted to maintain activated clotting time 180–220 s, activated partial thromboplastin time between 40 and 55 s, or anti-Xa activity between 0.2 and 0.3 IU per milliliter. This dose can be adjusted based on bleeding or thrombotic risk. Ventilator management during ECMO support includes minimal “rest” settings with a low rate, long inspiratory time, plateau pressure <25 cm H_2_O, low fraction of inspired oxygen <0.4, and positive end-expiratory pressure (PEEP) set at an appropriate level for patient condition (26).

### Statistical Analysis

Data were expressed as median (first and third quartiles) for non-normally distributed variables and number (percentage) for categorical variables. The normality of the data distribution was examined using the Kolmogorov–Smirnov tests. Baseline characteristics and outcomes were compared between VV ECMO patients and VA ECMO patients using the Mann–Whitney *U*-Test and Chi-squared/Fisher's exact test to detect any differences in the continuous and categorical variables, respectively. Data management and statistical analyses were conducted using STATA 15.1 SE (College Station, Texas 77845, USA).

## Results

### Patient Demographic and Pre-ECMO Characteristics

A total of 94 patients with infection-associated severe PARDS were enrolled and received ECMO support, with a medium length of hospital stay of 29 [Interquartile range (IQR) 20–47] days. Among them, 27.66% (26 cases) of patients were initially treated with VV ECMO, and 72.34% (68 cases) were placed on VA ECMO. Over the study period, increased utilization of VV ECMO trend was noted, from 24.44% (2019), to 30.00% (2020), to 45.45% (June 2021). The proportion of VV ECMO in 2018 was not included in the comparison because it was only 1 month in 2018 in our study period. The baseline and clinical characteristics of patients are shown in Table 1. The median age of all patients was 42 (IQR = 33–62) months, and the majority were male (*n* = 57, 60.64%). The most frequent infection site was pulmonary (*n* = 88, 93.62%), and the most common pathogens was bacterial-virus coinfection (*n* = 42, 44.68%), followed by bacterial (*n* = 24, 25.53%) and virus (*n* = 24, 25.53%).

**Table 1:** Baseline and clinical characteristics of patients (VA ECMO vs. VV ECMO).

	**Total (*n* = 94)**	**VA ECMO (*n* = 68)**	**VV ECMO (*n* = 26)**	***P*-value**
**Male sex**, ***n*** **(%)**	57 (60.64)	39 (57.35)	18 (69.23)	0.292
**Age, months**	42 (33.62)	38 (33.65)	59 (35.101)	0.061
**Age groups**				**0.006**
2–6 years	72 (76.60)	58 (85.29)	14 (53.85)	
7–10 years	11 (11.70)	5 (7.35)	6 (23.08)	
>10 years	11 (11.70)	5 (7.35)	6 (23.08)	
**Comorbidities**	11 (11.70)	9 (13.24)	2 (7.69)	0.455
Immunocompromised, *n* (%)	4 (4.26)	3 (4.41)	1 (3.85)	0.903
Leukemia and lymphoma, *n* (%)	7 (7.45)	6 (8.82)	1 (3.85)	0.411
**PARDS induced infection site**				
Pulmonary infection, *n* (%)	88 (93.62)	62 (91.18)	26 (100)	
**PARDS induced infection pathogen**				
Bacterial, *n* (%)	24 (25.53)	18 (26.47)	6 (23.08)	0.736
Viral, *n* (%)	24 (25.53)	22 (32.35)	2 (7.69)	**0.014**
Mycoplasma, *n* (%)	4 (4.26)	3 (4.41)	1 (3.85)	0.903
Bacterial-virus coinfection, *n* (%)	42 (44.68)	25 (36.76)	17 (65.38)	**0.013**
**Organ dysfunction**				
Acute renal injury, *n* (%)	16 (17.02)	10 (14.71)	6 (23.08)	0.334
Acute liver injury, *n* (%)	24 (25.53)	20 (29.41)	4 (15.38)	0.163
Left ventricular ejection fraction, (%)	66 (62.69)	68 (63.69)	65 (61.66)	0.164
Serum creatinine, μmol/L	30 (24.41)	30 (21.41)	31 (26.52)	0.203
**Pre-ECMO rescue therapy**				
NM blockade agents, *n* (%)	55 (58.51)	37 (54.41)	18 (69.23)	0.192
Inhaled nitric oxide, *n* (%)	14 (14.89)	10 (14.71)	4 (15.38)	0.934
Vasoactive drugs requirement, *n* (%)	71 (75.53)	52 (76.47)	19 (73.08)	0.732
PPV, *n* (%)	26 (27.66)	16 (23.53)	10 (38.46)	0.148
CRRT, *n* (%)	27 (28.72)	23 (33.82)	4 (15.38)	0.077
High-frequency oscillation ventilation, *n* (%)	12 (12.77)	7 (10.29)	5 (19.23%)	0.246
**Pre-ECMO ventilator settings**				
PaO_2_/FiO_2_, mmHg	56 (43.68)	55 (40.68)	59 (48.69)	0.149
Oxygen Index	34 (27.47)	36 (29.48)	29 (23.38)	**0.011**
Maximum FiO_2_, (%)	95 (90,100)	96 (90,100)	90 (90,100)	0.329
PEEP, cmH_2_O	10 (8.13)	10 (9.13)	10 (8.12)	0.150
Plateau pressure, cmH_2_O	30 (27.35)	31 (27.35)	30 (28.34)	0.929
MAP, cmH_2_O	19 (17.22)	20 (18.22)	18 (16.21)	0.106
**Pre-ECMO blood gas**				
pH	7.33 (7.23, 7.42)	7.32 (7.22, 7.42)	7.34 (7.28, 7.42)	0.439
PaCO_2_, mmHg	55 (42.69)	55 (43.71)	54 (42.59)	0.713
Plasma lactate, mmol/L	1.1 (0.7, 2.1)	1.1 (0.7, 2.2)	1.2 (0.7, 1.9)	0.770
**Time interval between mechanical ventilation and ECMO, hours**	65 (25.120)	88 (26.129)	29 (12.92)	**0.009**
**Severity at ECMO initiation**				
PRISM III	14 (10.18)	14 (11.18)	14 (7.18)	0.347
SOFA score	8 (5.9)	8 (6.10)	7 (5.8)	0.077
VIS	10 (0.35)	10 (0.50)	6 (0.15)	0.251
PIM3, (%)	15.94 (10.14, 26.83)	18.59 (10.26, 29.43)	11.97 (9.09, 21.49)	0.169
**Pneumothorax pre-ECMO**, ***n*** **(%)**	6 (18.18)	3 (13.04)	3 (30.00)	0.246

*Data are presented as median (IQR) or n (%). Significant values are showing in bold*.

*ECMO, extracorporeal membrane oxygenation; VA, venoarterial; VV, venovenous; PARDS, pediatric acute respiratory distress syndrome; PPV, prone position ventilation; CRRT, continuous renal replacement therapy; PaO_2_, partial pressure of oxygen; FiO_2_, fraction of inspired oxygen; PEEP, positive end-expiratory pressure; MAP, mean airway pressure; PH, potential of hydrogenpotential of hydrogen; PaCO_2_, partial pressure of carbon dioxide; PRISM III, Pediatric Risk of Mortality III score; SOFA, Sequential Organ Failure Assessment; VIS, Vasoactive-Inotropic Score; PIM3, Pediatric Index of Mortality 3*.

VA ECMO patients displayed a significantly higher proportion of children aged 2–6 years (85.29 vs. 53.85%, *p* = 0.006), viral-related PARDS (32.35 vs. 7.69%, *p* = 0.014), OI before cannulation (36 vs. 29, *p* = 0.011), and the longer time interval between MV and ECMO (88 vs. 29 h, *p* = 0.009) as compared with VV ECMO patients. By contrast, VV ECMO patients have a significantly higher proportion of bacterial-virus coinfection PARDS (65.38 vs. 36.76%, *p* = 0.013) and children aged >10 years (23.08 vs. 7.35%, *p* = 0.006) than VA ECMO patients. No significant intergroup differences were observed for comorbidities, pre-ECMO rescue therapy, blood gas, PRISM III, SOFA score, PIM3, VIS, and maximum FiO_2_.

Twelve (12.77%) patients were managed with high-frequency oscillatory ventilation (HFOV) prior to ECMO, 7 in the VA group and 5 in the VV group. Continuous renal replacement therapy (CRRT) was used in 27 (28.7%) patients. No significant differences between VV and VA groups were observed for serum creatinine, acute renal injury, left ventricular ejection fraction (LVEF), and the proportion of patients who received CRRT.

### Cannulation Strategies

Fifty-nine (86.76%) VA ECMO patients received surgical cut-down cannulation performed via neck vessels (right internal jugular vein-right carotid artery), with percutaneous femoral cannulation (femoral vein-femoral artery) performed in nine children. By contrast, most VV ECMO (*n* = 21, 80.77%) patients underwent percutaneous cannulation, using a modified Seldinger technique with imaging guidance by pediatric intensivists at the bedside in PICUs, while the other five cases received surgical cut-down cannulation. The right internal jugular vein-femoral vein was used as vascular access for 18 VV ECMO patients, as well as eight VV ECMO cases via femoral veins; Table 2).

**Table 2:** ECMO-related parameters (VA ECMO vs. VV ECMO).

	**Total (*n* = 94)**	**VA ECMO (*n* = 68)**	**VV ECMO (*n* = 26)**	***P*-value**
**Blood flow, ml/kg/min**	82 (67.100)	81 (68.100)	84 (67.95)	0.985
**Gas flow, L/min**	2.0 (1.0, 3.0)	2.0 (0.8, 2.5)	2.5 (2.0, 3.0)	**0.001**
**Cannulation configuration**				
Surgical open catheterization, *n* (%)	64 (68.09)	59 (86.76)	5 (19.23)	**<0.001**
Percutaneous cannulation, *n* (%)	30 (31.91)	9 (13.24)	21 (80.77)	**<0.001**

*Data are presented as median (IQR) or n (%). Significant values are showing in bold*.

*ECMO, extracorporeal membrane oxygenation; VA, venoarterial; VV, venovenous*.

### Outcomes

At the time of analysis, death occurred in 41 (43.62%) patients, which included 24 (58.54%) intractable respiratory failure, 9 (21.95%) circulatory failure, 5 (12.20%) infection and 3 (7.32%) massive hemorrhage (Table 3). VV ECMO was associated with a significantly lower in-hospital mortality (26.92 vs. 50.00%, *p* = 0.044), shorter duration of ECMO (158 vs. 202 h, *p* = 0.034) and MV (238 vs. 359 h, *p* = 0.007) compared with VA ECMO (Table 4). Successful weaning from ECMO was more common in VV ECMO than VA ECMO (73.08 vs. 55.88%) but without a significant difference (*p* = 0.127). Of note, no ECMO-related-neurologic complication (including infarction and hemorrhage) was seen in VV ECMO patients, while such complication occurred in 12 VA ECMO patients (0 vs. 17.65%, *p* = 0.022). The bleeding and bloodstream infection rates were similar between VV and VA ECMO patients. Likewise, the hospitalization costs [Chinese Yuan (CNY) 284,691 vs. 295,772, *p* = 0.649] and the length of stay (both PICU and hospital) were similar between VV and VA ECMO groups. However, VV ECMO patients were more likely to have oxygenator failure (15.38 vs. 2.94%, *p* = 0.027) and circuit component clots (23.08 vs. 7.35%, *p* = 0.034) than VA ECMO patients. In sensitivity analysis, the 1-month mortality post ECMO weaning and in-hospital mortality were identical (26.92% in VV ECMO patients vs. 50.00% in VA ECMO patients, *p* = 0.044) (Table 4). Furthermore, VV ECMO patients had lower 3-month mortality post ECMO weaning than VA ECMO patients (30.77 vs. 52.94%), while there was no significant difference (*p* = 0.054).

**Table 3:** Causes of death (VA ECMO vs. VV ECMO).

	**Total (*n* = 41)**	**VA-ECMO (*n* = 34)**	**VV-ECMO (*n* = 7)**	***P*-value**
**Causes of death**				
Hemorrhage, *n* (%)	3 (7.32)	2 (5.88)	1 (14.29)	0.437
Infection, *n* (%)	5 (12.20)	4 (11.76)	1 (14.29)	0.853
Intractable respiratory failure, *n* (%)	24 (58.54)	21 (61.76)	3 (42.86)	0.355
Circulatory failure, *n* (%)	9 (21.95)	7 (20.59)	2 (28.57)	0.642

*Data are presented as n (%)*.

*ECMO, extracorporeal membrane oxygenation; VA, venoarterial; VV, venovenous*.

**Table 4:** Outcomes and complications (VA ECMO vs. VV ECMO).

	**Total (*n* = 94)**	**VA ECMO (*n* = 68)**	**VV ECMO (*n* = 26)**	***P*-value**
**In-hospital mortality**, ***n*** **(%)**	41 (43.62)	34 (50.00)	7 (26.92)	**0.044**
**1-month mortality post ECMO weaning**, ***n*** **(%)**	41 (43.62)	34 (50.00)	7 (26.92)	**0.044**
**3-month mortality post ECMO weaning**, ***n*** **(%)**	44 (46.81)	36 (52.94)	8 (30.77)	0.054
**Duration time, hours**				
ECMO	176 (122,276)	202 (130,328)	158 (92,232)	**0.034**
Mechanical ventilation	330 (236,499)	359 (275,540)	238 (131,363)	**0.007**
**ECMO weaning rate**, ***n*** **(%)**	57 (60.64)	38 (55.88)	19 (73.08)	0.127
**ECMO related medical complication**				
Neurologic complications (infarction and hemorrhage), *n* (%)	12 (12.77)	12 (17.65)	0	**0.022**
Blood stream infections, *n* (%)	11 (11.70)	8 (11.76)	3 (11.54)	0.976
Massive bleeding, *n* (%)	21 (22.34)	16 (23.53)	5 (19.23)	0.654
**ECMO related device complication**				
Oxygenator failure, *n* (%)	6 (6.38)	2 (2.94)	4 (15.38)	**0.027**
Pump dysfunction, *n* (%)	2 (2.13)	2 (2.94)	0	0.377
Circuit component clots, *n* (%)	11 (11.70)	5 (7.35)	6 (23.08)	**0.034**
**Length of stay, day**				
PICU	20 (15.30)	22 (17.30)	15 (13.33)	0.130
Hospital	29 (20.47)	29 (23.50)	26 (18.38)	0.203
**Hospitalization costs, CNY**	295,154 (223,858, 432,817)	295,772 (209,372, 385,268)	284,691 (236,719, 432,817)	0.649

*Data are presented as median (IQR) or n (%). Significant values are showing in bold*.

*ECMO, extracorporeal membrane oxygenation; VA, venoarterial; VV, venovenous; PICU, pediatric intensive care medicine; CNY, Chinese Yuan*.

## Discussion

In this prospective multicenter cohort study of 94 patients with severe infection-associated PARDS, VV ECMO was associated with a significantly lower in-hospital mortality and less neurologic complications, shorter duration of ECMO and MV compared with VA ECMO. Over the study period, the proportion of initial VV ECMO therapy for this patient cohort increased steadily and now approaches 57.14%. In addition, the rates of bleeding, bloodstream infection, and successful ECMO weaning, hospitalization costs, and length of stay were similar between VV and VA ECMO groups. However, oxygenator failure was noted more frequently in VV than VA ECMO group. To our knowledge, this is the first prospective multicentral study to assess the difference between VV and VA ECMO in efficacy and safety for infection-associated severe PARDS.

ECMO has been used since the 1970s to improve gas exchange for children with severe acute respiratory failure who failed MV (27). There is level 1 evidence to support the use of ECMO for respiratory failure in neonatal and adults, but the evidence in pediatric respiratory failure remains less definitive (no level I evidence) (28–30). Based on the recent ELSO registry report, the survival rate to discharge for children with respiratory failure has been consistent over the past decade and was similar to our study (56% in our study vs. 60% in ELSO) (31). In addition, the OI before cannulation (36 in our study vs. 44 in ELSO) and the time interval between MV and ECMO (2.71 days in our study vs. 2 days in ELSO) were also consistent.

One retrospective review of the ELSO registry showed the proportion of initial VV ECMO support for adult ARDS patients significantly increased to 86% over the study period, and VV ECMO was associated with a higher rate of survival to discharge (19). In concordance with the result of the ELSO registry, we found the increasing trend of VV ECMO usage from 2019 to June 2021 (24.44–45.45%), and VV ECMO was associated with lower in-hospital mortality. The upward trend may be easily explained because many studies reported that VV ECMO was associated with better survival outcomes than conventional mechanical ventilation in patients with severe ARDS (32). Furthermore, in recent years, VV ECMO has been gradually expanded to the tertiary children's specialized hospitals in China due to technological advances. However, Jaber et al.'s (33) study for pediatric respiratory failure did not support our findings regarding the efficacy of V-V ECMO, with no statistically significant difference in survival to hospital discharge between VV ECMO and VA ECMO. Besma et al. (33) included patients with different primary indications for ECMO (including lung parenchymal disease, aspiration, and status asthmaticus); it is hard to tell whether the VV ECMO has a real effect in infection-associated PARDS. Our multicenter study is the first to recruit only infection-associated severe PARDS patients and identified the ideal ECMO modality.

Nevertheless, choosing between VV and VA support in patients with severe ARDS may be challenging because ARDS patients are likely to be complicated with rapidly evolving cardiovascular failure. In EOLIA clinical trials, 78% of the ARDS patients had severe sepsis or septic shock, and 74% had received vasopressors pre-ECMO (21). Consistent with EOLIA trials, our results showed high pre-ECMO inotropic agents/vasopressors requirements (76%) in infection-associated severe PARDS patients. The specific question of what to do with patients with primary respiratory failure and considerable hemodynamic compromise remains poorly addressed. However, studies in neonatal have noted that once adequate oxygenation is applied with VV ECMO and high pressures from MV are reduced, the need for vasoactive medications often disappears (34). According to the 2020 pediatric respiratory ELSO guideline, VV ECMO is recommended as an initial strategy for patients requiring minimal to modest inotropic/vasopressor support at the time of cannulation. That is because providing adequately O_2_ can sometimes result in improvement of hemodynamics such that VA ECMO will not be required (35).

During ECMO, neurological complications (including central nervous system hemorrhage or infarction) are common, potentially devastating and associated with increased mortality. In pediatric ECMO patients, neurological complications were noted in more than 10% of cases (36). In concordance with the result of the previous study, we found that the percentage of neurological complications was 13% in total patients. Delius et al. (17) compared neonatal patients supported with VV ECMO and VA ECMO for respiratory failure and found a lower rate of neurologic complications in VV ECMO patients. We also noted a lower rate of neurological complications in VV ECMO compared with VA ECMO patients (no patients vs. 17.65%, *P* = 0.002). In our study, most VA ECMO patients received cannulation via the carotid artery, which was similar to the result of the ELSO registry (16). Alteration of cerebral hemodynamics following carotid cannulation has been proposed as a mechanism of neurological complications (37, 38). However, no significant difference was identified in neurological complications between carotid and femoral cannulation for VA ECMO (16). In addition, Chenouard et al. (39) did not find evidence for an association between carotid cannulation and acute neurologic event, including hemorrhage. Furthermore, the percentage of neurological complications on VA ECMO observed here was 17%, lower than reported previously during pediatric ECMO for other indications (15, 39), suggesting that the carotid artery cannulation may not be associated with neurologic injury in PARDS. Nevertheless, additional prospective studies should be encouraged to compare the effects of cannulation via the carotid artery on neurological events in PARDS requiring ECMO support. Consequently, a higher rate of neurological complications could have contributed to the decreased survival associated with VA ECMO. Notably, using VV ECMO, subsequent modern trials have also demonstrated excellent outcomes that have reinvigorated its interest and use (30).

In our study, the bleeding and bloodstream infection rates were similar between VV and VA ECMO groups. However, oxygenator failure was more frequent in VV ECMO patients, consistent with previous literature (40). Common causes of oxygenator failure include thrombosis of the membrane due to inadequate anticoagulation, thrombocytosis and procoagulant states. Reasons for this observation will remain speculative, although it can be postulated that more frequent thrombosis of oxygenator in VV ECMO patients. Oxygenator thrombosis is reported in around 10–16% of patients, depending on the patient's age (41, 42). In our study, VA ECMO patients displayed a significantly higher proportion of younger children (aged 2–6 years), suggesting age may be associated with oxygenator thrombosis. Furthermore, a higher number of VV ECMO patients required circuit change attributed to coagulation-associated issues (including oxygenator failure) based on the ELSO Registry (43). However, we could not identify any risk factor of thrombosis due to the small sample size. Therefore, further studies should investigate the risk factor of thrombosis in ECMO-supported PARDS patients.

The duration of VV ECMO was shorter than VA ECMO in our study. This result may be explained because the OI was higher in the VA ECMO patients. The higher OI of VA ECMO patients is also linked to the longer duration of MV than VV ECMO patients. Furthermore, VA ECMO patients displayed a significantly higher proportion of viral-related PARDS than VV ECMO patients. The ECMO run time of viral pneumonia in pediatric patients was longer than bacterial pneumonia (44), suggesting the pathogen might affect the ECMO run time. However, there is a lack of studies to compare the duration of ECMO between different pathogens in PARDS.

There are several limitations to this study. First, we excluded patients <2 years of age due to the lack of double-lumen cannulas for VV ECMO in China. Therefore, the number of participants in this preliminary study is small, the value of our results is reduced, and it requires confirmation by a large dataset, particularly from the ELSO Registry. Moreover, lower OI before ECMO cannulation was noted in VV ECMO patients. So, it is unclear if the outcome is improved because the patients are “less sick.” In addition, hospital volume is not available in the present study. Some hospitals treating only a few patients per year are likely less experienced than others. Future studies should link outcomes with center experience and size. Another potential limitation is that some patients might have been excluded due to stringent exclusion criteria, limiting study result generalizability. There are no published guidelines for initiating ECMO in PARDS, particularly no clearly defined inclusion and exclusion criteria. Nevertheless, the study published in 2015 by PALICC provides some insight: the mortality of nearly 40% for OI > 16 (severe PARDS) was derived from the Children's Hospital of Los Angeles (CHLA) dataset and validated with the Australia New Zealand Intensive Care Society (ANZICS) dataset (1). Similarly, the mortality for children with respiratory failure requiring ECMO support was 40% based on the recent ELSO registry report (14), suggesting that an OI of 16 may be a suitable cut-off value for ECMO initiation in PARDS (28). In comparison with eligibility criteria of other studies, we enrolled patients with more stringent eligibility requirements: not only fulfilled the definition of severe ARDS with both PALICC (OI ≥ 16) and Berlin criteria (PaO_2_/FiO_2_ <100 mmHg) but also met the ECMO to Rescue Lung Injury in Severe ARDS (EOLIA) criteria for initiation of ECMO. Therefore, the patients in our study might have a higher level of disease severity in terms of hypoxemia than the populations in other studies that enrolled patients using PALICC or Berlin criteria alone. Finally, follow-up and long-term survival data are not yet available from this analysis. Despite these limitations, we believe the conclusion of the study would not be overturned.

These positive findings showed the conferred survival advantage and safety of VV ECMO compared with VA ECMO, suggesting that VV ECMO may be an effective initial treatment for patients with infection-associated severe PARDS, and VA ECMO should be reserved for conversion for refractory hypotension.

## Data Availability Statement

The raw data supporting the conclusions of this article will be made available by the authors, without undue reservation.

## Ethics Statement

The studies involving human participants were reviewed and approved by the Ethics Committee of Shanghai Children's Hospital (2018R052). The patients/participants provided their written informed consent to participate in this study.

## Author Contributions

YuC contributed to conceptualization and methodology. YZ contributed to data collection and editing of the manuscript. JD, JS, ZheZ, and DZ contributed to data collection. ZhenZ, YiC, CC, XQ, and XZ contributed to statistical analysis. WH contributed to editing and reviewing of the manuscript. All authors have read and approved the manuscript.

## Funding

This study was supported by Clinical Research Project of Shanghai Municipal Health Commission [grant number 202040467] and Shanghai Science and Technology Commission 2021 “Science and Technology Innovation Action Plan” Medical Innovation Research Special Project [grant numbers 21Y11902600 and 20Y11901300].

## Conflict of Interest

The authors declare that the research was conducted in the absence of any commercial or financial relationships that could be construed as a potential conflict of interest.

## Publisher's Note

All claims expressed in this article are solely those of the authors and do not necessarily represent those of their affiliated organizations, or those of the publisher, the editors and the reviewers. Any product that may be evaluated in this article, or claim that may be made by its manufacturer, is not guaranteed or endorsed by the publisher.
